# Comparison of percent density from raw and processed full-field digital mammography data

**DOI:** 10.1186/bcr3372

**Published:** 2013-01-04

**Authors:** Celine M Vachon, Erin EE Fowler, Gail Tiffenberg, Christopher G Scott, V Shane Pankratz, Thomas A Sellers, John J Heine

**Affiliations:** 1 Mayo Clinic College of Medicine, Department of Health Sciences Research, 200 First Street SW, Rochester, MN 55905, USA; 2 H. Lee Moffitt Cancer Center & Research Institute, Division of Population Sciences, 12902 Magnolia Drive, Tampa, FL 33612, USA

## Abstract

**Introduction:**

Mammographic density has been established as a strong risk factor for breast cancer, primarily using digitized film mammograms. Full-field digital mammography (FFDM) is replacing film mammography, has different properties than film, and provides both raw and processed clinical display representation images. We evaluated and compared FFDM raw and processed breast density measures and their associations with breast cancer.

**Methods:**

A case-control study of 180 cases and 180 controls matched by age, postmenopausal hormone use, and screening history was conducted. Mammograms were acquired from a General Electric Senographe 2000D FFDM unit. Percent density (PD) was assessed for each FFDM representation using the operator-assisted Cumulus method. Reproducibility within image type (*n *= 80) was assessed using Lin's concordance correlation coefficient (r_c_). Correlation of PD between image representations (*n *= 360) was evaluated using Pearson's correlation coefficient (r) on the continuous measures and the weighted kappa statistic (κ) for quartiles. Conditional logistic regression was used to estimate odds ratios (ORs) for the PD and breast cancer associations for both image representations with 95% confidence intervals. The area under the receiver operating characteristic curve (AUC) was used to assess the discriminatory accuracy.

**Results:**

Percent density from the two representations provided similar intra-reader reproducibility (r_c_= 0.92 for raw and r_c_= 0.87 for processed images) and was correlated (r = 0.82 and κ = 0.64). When controlling for body mass index, the associations of quartiles of PD with breast cancer and discriminatory accuracy were similar for the raw (OR: 1.0 (ref.), 2.6 (1.2 to 5.4), 3.1 (1.4 to 6.8), 4.7 (2.1 to 10.6); AUC = 0.63) and processed representations (OR: 1.0 (ref.), 2.2 (1.1 to 4.1), 2.2 (1.1 to 4.4), 3.1 (1.5 to 6.6); AUC = 0.64).

**Conclusions:**

Percent density measured with an operator-assisted method from raw and processed FFDM images is reproducible and correlated. Both percent density measures provide similar associations with breast cancer.

## Introduction

Increased mammographic breast density is an established breast cancer risk factor [1-3]. Irrespective of the method of measurement, the majority of studies have found a three- to sixfold increased risk of breast cancer in the highest vs. lowest density categories [2]. The majority of these studies estimated density from digitized film mammograms. Currently, more than 85 percent of the Mammography Quality Standards Act certified facilities operate FFDM units [4]. If breast density is to be used in the clinic for risk assessment and patient management, it is important to evaluate the performance of density estimated on full-field digital mammography (FFDM).

Film mammography and FFDM have similar diagnostic accuracy in breast cancer screening [5]. However, images acquired from these two forms of mammography may not appear similar on display [6]. FFDM systems can produce images in both the raw and clinical display representations, and these display formats differ across manufacturers. Clinical display images are processed (that is, enhanced) with algorithms developed by the respective manufacturers for improved diagnostic capability. Due to the size of images and storage considerations, the raw data is often discarded leaving only this processed data available for examination. Display variations may influence operator-assisted measurements of breast density. Currently, it is not known which FFDM representation is appropriate for percent density estimation.

In this report, we investigated the intra-measure reproducibility and the inter-measure correlation of percent density measures (PD) between raw and processed FFDM data representations. We also estimated and compared the associations of PD from both the raw and processed representations with breast cancer.

## Materials and methods

### Study design and population

We used a matched case-control design to examine associations between percent density from FFDM and breast cancer. The study population and data collection methods were described previously [7]. Briefly, cases were ascertained prospectively and retrospectively from patients with primary unilateral breast cancer attending the breast clinics at the H. Lee Moffitt Cancer Center between September 2007 and July 2011 (*n *= 180). Retrospective cases were selected from a record review of patients with breast cancer who received a four-view screening mammogram on the study FFDM unit at H. Lee Moffitt Cancer Center prior to or at the time of their breast cancer diagnosis. Potential prospective case patients were either Center breast screening patients without mammograms from the study unit or screening patients from the surrounding practices visiting the Center for diagnostic purposes. All retrospective cases with existing FFDM images on the study unit with primary unilateral breast cancer were selected. Prospective cases were recruited and offered a regular four-view (screening) mammogram from the study FFDM unit at the time of diagnosis.

Each case was classified into one of three screening-history categories referenced to the date of their study or most current FFDM mammogram prior to or at the time of diagnosis, for control matching purposes: women that had a history of normal screening 1) within 30 months prior to their study mammogram (*n *= 162), 2) outside of the 30-month window prior to their study mammogram (*n *= 13), or 3) women attending their initial screening mammogram (that is, no screening history) (*n *= 5). In all categories, the mammogram closest to diagnosis (including a mammogram at or immediately after diagnosis but before treatment) of breast cancer was ascertained for the study image. We selected the non-cancerous breast image for examination to avoid cancer present on the images, which could skew the density measure. Controls without a history of breast cancer (*n *= 180) who had archived four-view screening images acquired with the study FFDM unit were retrospectively selected from the pool of women undergoing screening mammography at H. Lee Moffitt Cancer Center between 2007 and 2011. Controls were individually matched to cases by age (± 2 years), postmenopausal hormone use and duration (that is, never used or former use and matched with ± 1 year duration of usage), study image breast side, and by the three screening-history categories defined above (that is, referenced relative to their study image date). For controls meeting the matching criteria, the most current mammogram on the study FFDM unit was used as the study image. Study data was collected under a protocol approved by the University of South Florida, Institutional Review Board (IRB number 104715D), Tampa, Florida. In accord with this approved protocol prospective case patients signed written informed consent, and retrospective data was collected from patients that signed information release documents previously.

### Acquisition of FFDM images and estimation of percent density

All images were acquired as standard screening, four-view mammograms. Craniocaudal (CC) views were used as the study images (that is, the non-cancerous CC view for cases and the matched side for the controls). All mammograms were acquired with one General Electric (GE) Senographe 2000D FFDM unit (General Electric Medical Systems, Milwaukee, WI, USA). This system produces data in both the raw and processed image representations, the latter for clinical display purposes. The raw images have 14-bit dynamic range for each pixel (that is, values raging from 0 to 16383), whereas the processed images have 12-bit dynamic range (that is, values ranging from 0 to 4095). The raw image pixel scale can be considered as the X-ray attenuation representation, where adipose image regions are bright (large pixel values) and fibroglandular regions are dark. The processed images have a reversed intensity scale (and reduced dynamic range) and appear similar to film mammograms. Image data was written to DVD storage in anonymous DICOM format from the GE workstation. This data was then uploaded to a UNIX-based server.

Percent density (PD) measurements were estimated from the raw (non-processed images) and processed representation FFDM images in DICOM format using the Cumulus3 software (University of Toronto). For a given representation, the dataset consisting of all cases and matched control images were de-identified and randomized. The reader (JH) was blinded to the case-control status and original image identifiers. When using Cumulus, the operator sets window levels and thresholds for each image to separate the dense from non-dense tissue and remove the off breast area region from the analysis. Percent density was calculated as the total dense area normalized by the total breast area to give the percentage of dense breast tissue as the PD measure.

### Statistical analyses

Patient characteristics and density measures were summarized as either the mean and standard deviation, or frequency and percentage, as appropriate. For each PD measure, quartiles were defined based on the distribution of that density measure among the control subjects for all applicable analyses (explicit quartile cutoff values and ranges are provided in the results below).

Eighty patient study images were selected at random from the controls to evaluate reproducibility. PD was measured by a single operator at two time points (separated on the order of weeks) to assess intra-measure PD reproducibility within a given data representation. Reproducibility was assessed using Lin's concordance correlation coefficient (r_c_), which measures the strength of the association between two measures about a line with an intercept of zero and a slope of one. We also applied linear regression analysis to evaluate the relationship between repeated measurements, which was summarized by the slope (m), intercept (b), and linear correlation coefficient (r).

The associations between the PD values estimated from the two data representations were assessed with a linear regression and summarized with m, b, and r. The distribution quartile concordance between the two PD measurements was evaluated with the weighted kappa statistic (κ). The combined case-control dataset was used for both the regression and κ analyses.

Conditional logistic regression was used to model and compare the association between quartile measures of PD, and breast cancer status, with the lowest quartile serving as the reference. Continuous measures (that is, standard deviations) of PD were also investigated with conditional logistic regression. The magnitudes of the associations were summarized by odds ratios (ORs) with 95% confidence intervals (CIs). The findings are presented for these models: (i) un-adjusted (ii) adjusted for body mass index (BMI) (kg/m^2^) only, (iii) adjusted for both BMI and breast area (or BA, measured in cm^2^), and (iv) adjusted for BMI, BA, and menopausal status (premenopausal if they reported experiencing menstrual cycles, otherwise postmenopausal). In all models, the ORs for BMI and BA are presented as per distribution (combined cases and controls) standard deviation increase. Similarly for the continuous PD measures, ORs are presented as per standard deviation increase for the respective FFDM representation. In all applicable models, we used premenopausal status as the reference for the menopause binary variable. Additionally as a secondary means of comparison, the area under the receiver operating characteristic curve (AUC) was computed as a summary of the ability of each model to discriminate between cases and controls. Methods for comparing correlated AUC [8] were used in this evaluation.

## Results

Cases and matched controls were of similar age and postmenopausal hormone use, as expected due to the matched design (Table 1). Cases had a higher BMI (26.6 vs. 25.3) and were more likely postmenopausal (79% vs. 73%) than controls. The mean PD was higher for cases than controls for both FFDM representations (*P *< 0.01 for the raw data and *P *< 0.03 for the processed data from the paired *t *test). Mean PD quantities were greater for the processed images than the raw images within cases (*P *< 0.11) and controls (*P *< 0.001), although differences were not significant among the cases (Table 1).

**Table 1:** Participant characteristics by case, control, and combined grouping.

Characteristic	Case n	Case Mean/SD^b^ or %	Control n	Control Mean/SD^b^ or %	Total n	Total Mean/SD^b^ or %	*P* ^d^
Age	180	58.60/10.46	180	58.53/10.40	360	58.56/10.41	0.232
Menopausal Status							0.049
Postmenopausal	142	78.89%	132	73.33%	274	76.11%	
Premenopausal	38	21.11%	48	26.67%	86	23.89%	
PMH^a^							0.297
Never-used	96	53.33%	101	56.11%	197	54.72%	
1 - 5 yrs	30	16.67%	27	15.00%	57	15.83%	
6 - 10 yrs	18	10.00%	19	10.56%	37	10.28%	
11 - 15 yrs	13	7.22%	9	5.00%	22	6.11%	
> 15 yrs	23	12.78%	24	13.33%	47	13.06%	
BMI (kg/m^2^)^c^	179	26.56/4.62	180	25.25/4.25	359	25.90/4.48	0.009
Breast area (cm^2^)	180	139.01/47.83	180	131.52/40.60	360	135.26/44.46	0.104
PD_proc _(%)	180	22.48/14.41	180	19.76/12.89	360	21.12/13.72	0.029
PD_raw _(%)	180	21.33/15.66	180	17.70/14.58	360	19.52/15.22	0.009

^a^Postmenopausal hormonal (PMH) by years (yrs); ^b^the mean and standard deviation (SD) for the raw and processed (proc) percentage of breast density measures (PD), age (years), body mass index (BMI kg/m^2^) and breast area (cm^2^) are also provided; ^c^BMI was missing for one case observation; ^d^*P *values (*P*) were determined with the paired *t *test (continuous variables) or the exact McNemar's test (binary variables) by comparing the respective case and control quantities.

**Figure 1:**
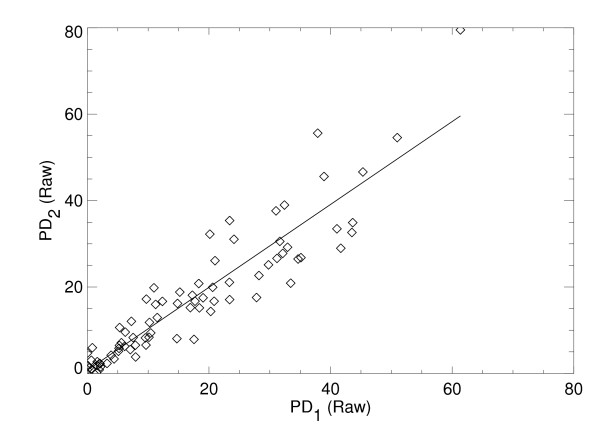
**Reproducibility of percent density from the raw image representation**. This shows the percent density measure (PD) applied to a sample of 80 raw images at two time points labeled as PD_1 _and PD_2_, respectively (diamonds) evaluated with this relationship PD_2 _= m ×PD_1 _+ b, where m and b are the slope and intercept. The fitted line (solid) was estimated with regression analysis giving: m = 0.96 ± 0.05, b = 0.76, and linear correlation = 0.92.

**Figure 2:**
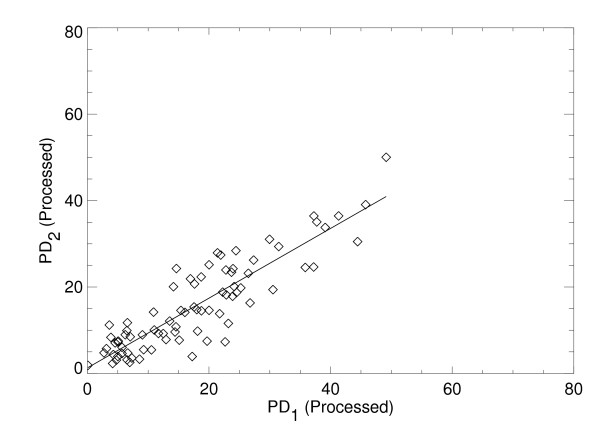
**Reproducibility of percent density from the processed image representation**. This shows the percent density measure (PD) applied to a sample of 80 processed images (same patient samples shown in Figure 1) at two time points labeled as PD_1 _and PD_2_, respectively (diamonds) evaluated with this relationship PD_2 _= m × PD_1 _+ b, where m and b are the slope and intercept. The fitted line (solid) was estimated with regression analysis giving: m = 0.81 ± 0.05, b = 1.3, and linear correlation = 0.89.

The intra-operator reproducibility for PD was high as evident from the concordance correlation of r_c _= 0.92 for the raw images, and r_c _= 0.87 for the processed images. The regression analyses from the repeated PD measurements also showed the degree of reproducibility with m = 0.96 ± 0.05, b = 0.76 and r = 0.92 for the raw images shown in Figure 1, and m = 0.81 ± 0.05, b = 1.3, and r = 0.89 for the processed images shown in Figure 2. The similarity between the respective r_c _and r indicates that the first and second PD reproducibility measurement distributions have close agreement in the mean and variance for a given image representation. However, the deviation in the processed PD from the ideal regression parameter values (that is, m = 1 and b = 0) indicates that PD is better reproduced from the raw images.

**Figure 3:**
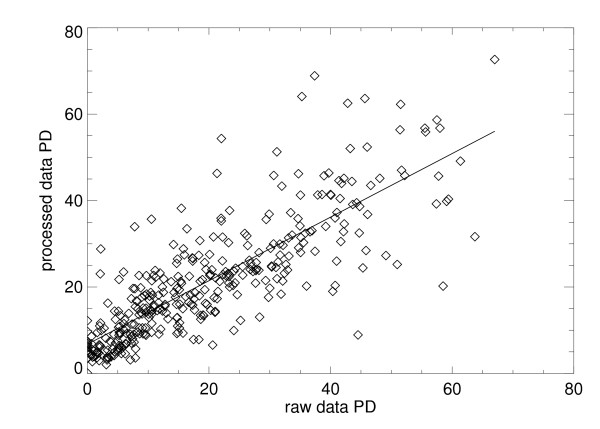
**Comparison of percent density from the raw and processed images**. This plot shows the relation between the percent density measure (PD) applied to the raw and processed data (diamonds) for the entire case-control dataset evaluated with this relationship PD_processed _= m × PD_raw _+ b, where m and b are the slope and intercept. The fitted regression line (solid) shows the processed PD as a linear function of the raw PD giving: m = 0.74 ± 0.03, b = 6.76, and linear correlation = 0.82.

The PD measurements from the processed clinical display images are linearly associated with the PD measurements from the raw images with r = 0.82. As shown in Figure 3, the processed PD is slightly elevated relative to the raw PD; the slope is significantly different from unity with m = 0.74 ± 0.03, and the intercept is significantly different from zero with b = 6.76. When categorizing each of the PD distributions into their respective quartiles (see Table 2 for cutoff values and ranges), the relationship between the two measurements shows moderate agreement, with a weighted kappa (κ) of 0.64 (95% CI: 0.59 to 0.70) [9]. The joint frequency quartile distribution for the two PD measures is provided and compared in Table 2. Of the 360 subjects, 217 (60.3%) were classified into the same quartiles by assessments made on their raw and processed images. Of the 143 with discrepant classifications, 131 (91.6%) differed by a single quartile (when applying the published Boyd categories [10], results were similar with 66% of the patients classified in the same category and 32.5% within one category shift, see Additional file 1).

**Table 2:** Joint frequency quartile distribution for the number of observations (n) per-quartile for the percent density measurements (PD) from the raw (vertical) and processed (horizontal) image representations.

	**PD (processed)**				
		
**PD (raw)**	**Quartile 1**	**Quartile 2**	**Quartile 3**	**Quartile 4**	**n**
	**[0.0, 9.2)**	**[9.2, 18.1)**	**[18.1, 26.3)**	**[26.3, 100)**	
	
Quartile 1	52	16	2^†^	1^†^	71
[0.0, 5.5)					
Quartile 2	19	51	21	3^†^	94
[5.5, 14.7)					
Quartile 3	3^†^	28	40	21	92
[14.7, 26.9)					
Quartile 4	1^†^	2^†^	26	74	103
[26.9, 100)					
	
n	75	97	89	99	360

PD quartile ranges and cutoff values (corresponding to those used in Tables 3 and 4) are provided under the quartile headings. ^†^Observations that experienced two inter-representation quartile shifts (that is, 12/360 or approximately 4% of the observations).

The PD and breast cancer associations for both FFDM representations are summarized in Table 3 (raw) and Table 4 (processed). The ORs and AUCs for the associations of breast cancer with categorical (quartiles of PD based on controls, provided in Table 2) and continuous measures of PD (per standard deviation) are provided. Because our interest is in the performance of the Cumulus method applied to FFDM image formats, we focus on the simple BMI-adjusted models for the primary comparison. The associations and discriminatory accuracy of quartiles of PD from raw (OR: 1.0 (ref.), 2.6 (1.2 to 5.4), 3.1 (1.4 to 6.8), 4.7 (2.1 to 10.6); AUC = 0.63) and processed images (OR: 1.0 (ref.), 2.2 (1.1 to 4.1), 2.2 (1.1 to 4.4), 3.1 (1.5 to 6.6); AUC = 0.64) with breast cancer were similar. The associations of continuous PD and breast cancer for the raw (OR = 1.8 (1.3 to 2.4); AUC = 0.64) and processed (OR = 1.6 (1.2 to 2.2); AUC = 0.63) images were also similar. In all of the BMI-adjusted models, the ORs corresponding to the PD and breast cancer associations were significant (Tables 3 and 4). The BMI variable had the largest influence on the breast density associations, as expected [11]. The addition of breast area and menopausal status together strengthened (marginally) the PD associations for both representations. In the fully adjusted continuous PD models, the association of breast cancer with percent density from raw (OR = 1.8 (1.3 to 2.4)) and processed images (OR = 1.7 (1.2 to 2.3)) showed little difference from the BMI-adjusted models.

**Table 3:** Associations of categorical and continuous percent density analysis from raw full-field digital mammography (FFDM) representation with breast cancer (*n *= 180 case and matched control pairs).

Quartile PD_raw_	Case *N *= 180	Unadjusted OR (95% CI)	BMI adjusted OR (95% CI)	BMI, BA adjusted OR (95% CI)	BMI, BA, menopause adjusted OR (95% CI)
1	26	1.00 (Ref.)	1.00 (Ref.)	1.00 (Ref.)	1.00 (Ref.)
2	49	2.21 (1.07, 4.55)	2.55 (1.20, 5.42)	2.60 (1.21, 5.55)	2.83 (1.30, 6.16)
3	47	2.17 (1.07, 4.40)	3.12 (1.44, 6.76)	3.15 (1.45, 6.84)	3.45 (1.55, 7.65)
4	58	2.70 (1.32, 5.49)	4.69 (2.08, 10.58)	4.85 (2.13, 11.07)	5.17 (2.24, 11.96)
BMI^a^		n/a	1.56 (1.22, 1.99)	1.49 (1.13, 1.97)	1.46 (1.10, 1.93)
BA^a^		n/a	n/a	1.09 (0.83, 1.44)	1.11 (0.84, 1.48)
Menopause^b^		n/a	n/a	n/a	2.42 (1.00, 5.86)
AUC^c^		0.568	0.631	0.634	0.641

Continuous PD_raw_		Unadjusted OR (95% CI)	BMI adjusted OR (95% CI)	BMI, BA adjusted OR (95% CI)	BMI, BA, menopause adjusted OR (95% CI)

PD^a^		1.38 (1.07, 1.77)	1.76 (1.31, 2.37)	1.79 (1.33, 2.43)	1.79 (1.32, 2.42)
BMI^a^		n/a	1.60 (1.25, 2.06)	1.52 (1.15, 2.01)	1.48 (1.12, 1.97)
BA^a^		n/a	n/a	1.12 (0.85, 1.48)	1.13 (0.85, 1.50)
Menopause^b^		n/a	n/a	n/a	2.06 (0.87, 4.89)
AUC^c^		0.571	0.638	0.641	0.655

^a^The ORs for BMI (kg/m^2^) and BA (cm^2^), and PD (continuous models) are cited in per standard deviation increase; ^b^for the binary menopausal variable, pre-menopausal status is the reference; ^c^AUC is a measure of discriminatory accuracy. AUC, area under the receiver operating characteristic curve; BA, breast area; BMI, body mass index; CI, confidence interval; PD, percent density; OR, odds ratio.

**Table 4:** Associations of categorical and continuous percent density analysis from processed (proc) full-field digital mammography (FFDM) representation with breast cancer (*n *= 180 case and matched control pairs).

Quartile PD_proc _	Case *N *= 180	Unadjusted OR (95% CI)	BMI adjusted OR (95% CI)	BMI, BA adjusted OR (95% CI)	BMI, BA, menopause adjusted OR (95% CI)
1	30	1.00 (Ref.)	1.00 (Ref.)	1.00 (Ref.)	1.00 (Ref.)
2	52	1.70 (0.93, 3.13)	2.16 (1.13, 4.12)	2.24 (1.16, 4.30)	2.59 (1.32, 5.09)
3	44	1.51 (0.81, 2.81)	2.22 (1.11, 4.44)	2.35 (1.16, 4.77)	2.70 (1.30, 5.62)
4	54	1.90 (0.98, 3.67)	3.14 (1.49, 6.60)	3.42 (1.59, 7.39)	3.99 (1.80, 8.84)
BMI^a^		n/a	1.50 (1.18, 1.90)	1.41 (1.08, 1.84)	1.39 (1.06, 1.81)
BA^a^		n/a	n/a	1.14 (0.86, 1.51)	1.18 (0.89, 1.57)
Menopause^b^		n/a	n/a	n/a	2.82 (1.16, 6.84)
AUC^c^		0.556	0.636	0.638	0.647

Continuous PD_proc_		Unadjusted OR (95% CI)	BMI adjusted OR (95% CI)	BMI, BA adjusted OR (95% CI)	BMI, BA, menopause adjusted OR (95% CI)

PD^a^		1.32 (1.02, 1.69)	1.63 (1.22, 2.18)	1.67 (1.24, 2.25)	1.68 (1.24, 2.28)
BMI^a^		n/a	1.53 (1.20, 1.94)	1.45 (1.10, 1.89)	1.42 (1.08, 1.86)
BA^a^		n/a	n/a	1.13 (0.85, 1.48)	1.14 (0.86, 1.51)
Menopause^b^		n/a	n/a	n/a	2.19 (0.93, 5.19)
AUC^c^		0.550	0.628	0.635	0.643

^a^The ORs for BMI (kg/m^2^) and BA (cm^2^), and PD (continuous models) are cited in per standard deviation increase; ^b^for the binary menopausal variable, pre-menopausal status is the reference; ^c^AUC is a measure of discriminatory accuracy. AUC, area under the receiver operating characteristic curve; BA, breast area; BMI, body mass index; CI, confidence interval; PD, percent density; OR, odds ratio.

Secondarily, we compared the discriminatory accuracy (or AUC) of the ability of various models to separate the cases from the controls (Tables 3 and 4). Within either representation for both quartile and continuous unadjusted models, the AUC increase due to the inclusion of BMI was significant (*P *< 0.003), whereas the addition of the other covariates with BMI individually, or simultaneously, produced marginal AUC increases (*P *> 0.20). However, there was no statistical significant difference in AUC for PD from raw vs. processed images with breast cancer for either the unadjusted or adjusted models (*P *> 0.30).

## Discussion

Several of our findings merit particular comment specific to this FFDM technology. First, PD was reproducible whether assessed from raw or processed images but reproducibility was slightly higher for the raw representation. Second, PD assessed from the two FFDM representations was highly correlated, although PD measured from the processed images was slightly greater than from the raw images. And, earlier analysis showed that PD estimated from processed images (same type of FFDM unit used here) was less than that estimated from film [12]. These findings suggest that when merging mammograms from film and FFDM systems for breast density analyses, the choice of both film vs. FFDM as well as FFDM representation could impact the study. Much of the discrepancy between the two image representations of PD was accounted for by a one quartile shift. However, a small proportion of samples, approximately 4% (that is 12/360), showed a large discrepancy of two quartile shifts (Table 2). Further, our inter-FFDM measure agreement is in the range of inter-rater PD agreement using various FFDM models [13]. We demonstrated high intra-rater reproducibility of both the raw and processed FFDM PD, which is consistent with findings by other groups using different types of FFDM units [14]. Finally, this work showed that PD estimated from either representation was associated with breast cancer.

The inter-FFDM representation differences in our findings may be in part due to the pixel dynamic range compression (14 to 12 bit) or the two-stage mapping (that is, the mapping applied by the manufacturer) used to form the processed images from the raw images [15]. The detector response is linear in X-ray exposure for this system over a wide range of energies [16], which may also influence the raw image display. These differences may also be due to operator preference or natural variation (that is, each image requires its own window level and threshold adjustments). Because there are relatively few FFDM reports evaluating PD from FFDM, the generality of our findings will require further investigations.

The present results should be interpreted in light of certain limitations and qualifications. Limitations of the study include the relatively limited number of cases and controls, emphasizing the importance of replicating these findings in larger studies. Also, the use of mammograms at the time of diagnosis limits our ability to draw additional conclusions regarding temporality and risk. And, we were not able to examine or adjust for the perimenopausal category within menopausal status, due to the limited nature of the question; however, this should not influence the comparison between the two density measures. These findings apply to one specific FFDM design and to a Cumulus operator with considerable experience and preferences. Therefore, it will be necessary to conduct similar research on other FFDM technologies with multiple operators and larger datasets to ensure our results generalize. Similarly, the operator was not a clinician, and our results strictly apply in the research environment and not to the clinical setting *per se*. Although given an equally skilled operator, we would expect the findings to theoretically translate to the clinic. The detector size for the FFDM design (that is, the earliest FFDM technology) used in our study precludes its use on women with large breasts with one exposure [6], limiting the range of breast sizes for the cases and controls included in the study [7] and consequently our generalizability to women with large breasts. Our findings will require replication on newer FFDM models that do not have the size limitation. We observed slightly higher reproducibility in the analysis of the raw data than processed data, but this may reflect that the operator has more experience labeling raw data than processed data rather than any true difference. The reproducibility analysis was restricted to control images, which should not limit generalization because there are not large differences in the unadjusted case-control PD distributions. Moreover from our operator's experience, the very low-density images, which are less prevalent in cases, are more difficult to label (that is, in these situations the operator may perceive a range of acceptable control settings before the density estimation is performed) than high-density images. The reproducibility analysis was performed over a short time period, which could result in higher reproducibility compared to a longer span between evaluations. Finally, we did not have corresponding mammograms from film mammography units to serve as comparison to the raw and processed FFDM representations.

The Cumulus software was developed for digitized film data applications, not for FFDM. The Cumulus3 operating instructions indicate that the input data scale should parallel that of the display representation images (that is, larger pixel values should correspond with radiographically dense or bright image regions). It should be noted, the pixel scale is reversed for our raw images (that is, larger values corresponded with fatty area). This suggests that the raw images from this specific mammography unit are in the incorrect format and will not display properly within the Cumulus3 interface. To the contrary, we did not experience any difficulty reading the raw images without additional preparation. This raw image pixel scale was automatically inverted (within Cumulus) so that adipose regions were dark and glandular regions bright when reading the raw images into the Cumulus environment. Because we are uncertain as to why the raw images displayed properly within the Cumulus3 interface, in conflict with operating instructions, this work should be replicated on both similar and different FFDM designs as well. Notwithstanding these artifacts, the comparisons and findings presented in this report are internally valid.

## Conclusions

In summary, PD from the two FFDM representations was reproducible and correlated. Further, the breast cancer associations were similar across the data representations and agree with those reported previously [2]. However, the raw data representation provided slightly better reproducibility. Thus, the raw data may be preferable for PD applications when possible. Although these results are encouraging, additional evaluation of prospective and larger study populations, operators, and system designs is required to confirm our findings.

## Abbreviations

AUC: the area under the receiver operating characteristic curve; b: intercept; BA: breast area; BMI: body mass index; CI: confidence interval; FFDM: full-field digital mammography; κ: weighted kappa statistic; m: slope; OR: odds ratio; PD: percent density or percentage of breast density measure; r: Pearson's correlation coefficient; r_c_: Lin's concordance correlation coefficient.

## Competing interests

The authors declare that they have no competing interests.

## Authors' contributions

The database was constructed by GT and EF under the supervision of JH. JH and CV developed the manuscript content. EF, CS, VP, and JH performed the data analysis. CV and TS provided the epidemiologic guidance. All authors contributed to manuscript composition. All authors read and approved the final manuscript.

## Supplementary Material

Additional file 1 **Table S1**. This table provides the joint frequency distribution for the number of observations (n) per-Boyd ^a^category for the percent density measurements (PD) from the raw (vertical) and processed (horizontal) image representations. Click here for file
