# Supplementary material for: Comparison of percent density from raw and processed full-field digital mammography data
Source: Breast Cancer Res. 2013 Jan 4;15(1):R1. doi: 10.1186/bcr3372 (PMC3672765; doi:10.1186/bcr3372)
Supplement: Additional file 1 — Table S1. This table provides the joint frequency distribution for the number of observations (n) per-Boyd acategory for the percent density measurements (PD) from the raw (vertical) and processed (horizontal) image representations. [file bcr3372-S1.DOC]

| PD (raw) | PD (processed) | | | | n |
| --- | --- | --- | --- | --- | --- |
|  | Category 1 | Category 2 | Category 3 | Category 4 |  |
|  | [0.0, 10) | [10, 25) | [25, 50) | ^a^[50, 75) |  |
| Category 1 | 78 | 43 | 2 | 0 | 123 |
| [0.0, 10) |  |  |  |  |  |
| Category 2 | 9 | 92 | 23 | 1 | 125 |
| [10, 25) |  |  |  |  |  |
| Category 3 | 1 | 26 | 61 | 7 | 95 |
| [25, 50) |  |  |  |  |  |
| Category 4 | 0 | 1 | 9 | 7 | 17 |
| ^a^[50, 75) |  |  |  |  |  |
| n | 88 | 162 | 95 | 15 | 360 |
|  |  |  |  |  |  |

^a^There were no samples in the 5^th^ category (the category for PD >75 is not included). Approximately 66% of the samples (n = 238) were in agreement, 32.5% (n = 117) differed by one category and 1.4% (n = 3) differed by 2 categories. The two measures were in agreement with κ = 0.62 (0.56, 0.68).
